# A Case in Practice

**Published:** 1871-07

**Authors:** H. Scott


### A CASE IN PRACTICE.

BY H. SCOTT.

Mr. R. called to consult me about a case of neuralgia which he said had been troubling him for some time. He said he had no unsound teeth that he could blame, but that he had spent a sleepless night. The pain, he said, seemed to be in both his jaws, and put his finger to the upper bicuspids on the right side, as the point of the greatest suffering. There were four or five small gold fillings in his mouth, besides one of amalgam; otherwise there was nothing visible that could lead to the suspicion of toothache. I examined the approximal surfaces of all his teeth, and found them intact. The sounding hammer was next used, which caused pain in the second right superior molar.

### HISTORY OF THE CASE.

Six months before, the tooth had been sore, with some tumefaction under the eye, and the patient underwent a three or four weeks course of alterative treatment for facial neuralgia, with no other recognized result than the simple baffling of science. From that time on he had suffered various kinds of harrassing pains in the jaws, ears and temples, and had taken large quantities of quinine and iron. The case had been submitted to two dentists, who could find nothing wrong with any of the teeth. At the time he called to see me, he had just come from his physician, and had in his pocket a professional prescription, which he left with me, but which I forbear to transcribe, further than to say that quinine was the governing ingredient.

### TREATMENT.

The investing membranes were too much inflamed to admit of cutting out the filling and perforating the nerve cavity, except at the expense of considerable pain, and besides, that course did not, in my judgment, promise a successful issue under all the circumstances. I therefore suggested the removal of the tooth, which was done at once. The filling was in the anterior fissure of the grinding surface, and had a small face.

### AUTOPSY.

Upon splitting the tooth with a hammer and chisel, the pulp cavity was found filled with a grumous fluid, the effluvium of which rendered the air of the room for a few seconds nearly irrespirable. The filling was narrow and deep, and had from the first impinged the membrane towards the labial surface of the tooth, and hence all the disasters which followed.

The single remark I feel inclined to submit at present is, that the remedial and healing arts seem not to be yet out of the wilderness.

